# Rhizosphere and Soil Depth Differentially Shape Microbial Community Composition and Assembly in *Phragmites australis* Salt-Marsh Soils

**DOI:** 10.3390/microorganisms14091891

**Published:** 2026-08-26

**Authors:** Lei Wang, Junzhe Shi, Liwen Li, Kaipeng Jiang, Jingwei Lian, Dezong Sui, Sian Liu, Yingdan Yuan, Yingzhou Tang

**Affiliations:** 1 Jiangsu Academy of Forestry, Nanjing 211153, China; lkywanglei@163.com (L.W.);; 2 Co-Innovation Center for Sustainable Forestry in Southern China, Jiangsu Province Key Laboratory of Soil and Water Conservation and Ecological Restoration, Nanjing Forestry University, Nanjing 210037, China; 3 College of Horticulture and Landscape Architecture, Yangzhou University, Yangzhou 225009, China

**Keywords:** *Phragmites australis*, coastal wetland, soil microorganisms, rhizosphere, soil layer

## Abstract

Common reed (*Phragmites australis*) is a native dominant plant in many coastal wetlands. To determine how rhizosphere effects and soil depth shape microbial communities, we sampled the rhizosphere and three bulk-soil layers (0–15, 15–30, and 30–45 cm) in a monodominant common-reed stand in a coastal salt marsh. Soil physicochemical properties and bacterial and fungal α-diversity, community composition, and assembly processes were evaluated using one-way ANOVA, principal coordinates analysis (PCoA), permutational multivariate analysis of variance (PERMANOVA), neutral community models, and phylogenetic null models. Rhizosphere pH was lower than that of 0–15 cm bulk soil (mean 8.434 vs. 8.712) but remained alkaline; soil organic matter, total nitrogen, hydrolyzable nitrogen, and total phosphorus were greatest in the rhizosphere. Neither bacterial nor fungal richness or Shannon diversity differed significantly among compartments (*p* > 0.05); fungal Shannon means ranged from 2.083 to 3.077, with relatively higher Bacteroidota and lower Acidobacteriota abundance in the rhizosphere. Fungal composition did not differ significantly (pseudo-F = 0.609, R^2^ = 0.102, *p* = 0.9112), although Mucoromycota and Rozellomycota were relatively more abundant in the rhizosphere. Phylogenetic null models indicated predominantly deterministic bacterial assembly, with 10% dispersal limitation in the 15–30 cm layer. Fungal assembly was predominantly stochastic in bulk soils, whereas the rhizosphere was an exception: heterogeneous selection accounted for 60% of pairwise comparisons and median βNTI exceeded +2. The Mantel test identified only the association between total phosphorus and bacterial diversity as significant (0.01 < *p* < 0.05). These results show that rhizosphere filtering strongly structured bacterial composition and imposed deterministic selection on rhizosphere fungi.

## 1. Introduction

Coastal wetlands are critical transition zones between terrestrial and marine ecosystems, featuring unique hydrological, soil, and biological community structures. They play irreplaceable ecological roles in maintaining biodiversity, purifying water bodies, regulating climate, and sequestering and storing carbon, making them vital components of global ecosystems [1]. In typical coastal salt-marsh vegetation zones, communities are often dominated by a single dominant species [2]. Common reed (*Phragmites australis*), the most widely distributed and highly adaptable native dominant plant in China’s coastal salt marshes, can tolerate harsh habitats such as high salinity and flooding. Through root activities, litter decomposition, and rhizosphere microenvironment regulation, it significantly alters soil physicochemical properties and profoundly shapes soil microbial community composition, serving as a key species for maintaining the stability of wetland soil ecosystems [1,3,4].

Current research on the effects of coastal wetland plants on soil microorganisms mostly focuses on comparisons between invasive and native plant communities or is limited to analyses of single soil layers or single rhizosphere compartments. Systematic studies on the differences in soil microbial communities across different soil depths and rhizosphere/non-rhizosphere microdomains for common reed remain relatively insufficient [3]. Clarifying the differences in diversity, community composition, and ecological assembly processes of soil microorganisms between rhizosphere and non-rhizosphere soils, as well as between surface and deeper layers in common-reed communities, will help reveal the regulatory mechanisms of native plants on wetland soil ecosystems, improve the theory of vertical distribution of underground biological communities in coastal wetlands, and provide scientific evidence for wetland ecological protection, native vegetation restoration, and soil health assessment [5,6,7].

In terms of horizontal differences between the rhizosphere and non-rhizosphere, common reed can actively construct a microenvironment distinctly different from the surrounding soil through the secretion of organic acids, sugars, amino acids, and other root exudates, along with oxygen release [3,8]. Previous studies have reported higher microbial α-diversity in the common-reed rhizosphere, potentially associated with continuous carbon inputs and relatively aerobic microsites; whether this expectation holds across rhizosphere and depth compartments remains uncertain [9]. In contrast, non-rhizosphere soil, lacking fresh organic matter replenishment and remaining in a long-term reduced state, has a relatively simpler microbial community structure and lower diversity [8,10]. More importantly, the common-reed rhizosphere may enrich functional groups such as nitrogen fixers, nitrifiers, and phosphate-solubilizing bacteria or fungi, which can contribute to nutrient transformations and plant nutrient availability [11]. In non-rhizosphere areas, anaerobic microorganisms such as sulfate-reducing bacteria account for a higher proportion, reflecting stronger reducing environmental characteristics [12,13].

In the vertical distribution across soil layers, the influence of common reed on microbial communities gradually weakens with increasing soil depth [14,15]. The 0–15 cm surface soil is the region where common-reed ecological effects are most concentrated. Here, root density is high, litter input is large, and oxygen supply is relatively abundant due to the aerenchyma tissue of common reed, forming a region with high microbial activity and rich diversity [16]. Microbial communities in this soil layer not only exhibit high diversity but also more complex and stable species co-occurrence networks, with tight interspecific collaborative relationships and greater ecological functional resilience [17]. The 15–30 cm middle soil layer serves as a transition zone. Although root density and oxygen supply decrease, common reed can still transport some oxygen through aerenchyma tissue to maintain microaerobic conditions [18,19]. Community assembly at this depth gradually shifts from resource-driven at the surface to environment-filtering dominated, with community composition more sensitive to physicochemical factors such as redox potential [20]. The 30–45 cm deep soil layer is far from the main root activity zone, with stable and highly anoxic conditions; the direct influence of common reed is weak, and the community is dominated by obligate anaerobes such as methanogenic archaea and iron/sulfate-reducing bacteria (20). However, common reed can slowly input recalcitrant organic matter through deep root systems, providing long-term but limited carbon sources for deep-layer microorganisms, thereby influencing community structure and function over longer timescales [21,22].

Based on this background, we compared the rhizosphere of common reed with three bulk-soil depth intervals (0–15, 15–30, and 30–45 cm). We quantified soil pH, SOM, TN, TP, TK, HN, AP, and AK; characterized bacterial and fungal α-diversity and community composition; and evaluated assembly using neutral community models and βNTI/RCbray-based null models. We tested two hypotheses: (1) rhizosphere effects would produce stronger changes in soil properties and microbial composition than soil depth alone, and (2) bacterial and fungal assembly processes would differ among the rhizosphere and depth compartments. The study aimed to resolve how rhizosphere effects and soil depth jointly shape microbial community composition and assembly in a coastal wetland.

## 2. Materials and Methods

### 2.1. Soil Sample Collection

A typical monodominant common-reed stand in the Yancheng coastal wetland, China (33°31′20″ N, 120°38′10″ E), was selected as the study site. Based on the ISRIC SoilGrids classification and the World Reference Base for Soil Resources (WRB), the soil at the sampling site was identified as a Fluvisol at the Reference Soil Group level. Common reed in this stand grew well, with no obvious human disturbance or invasion by exotic species. Three 10 m × 10 m quadrats separated by >5 m were established within the stand. Within each quadrat, samples were collected using a five-point design comprising one central and four peripheral points. For each compartment, subsamples from the corresponding point position in the three quadrats were pooled to form one composite field replicate. The five point positions therefore yielded five composite replicates for each of the four compartments—the rhizosphere and the 0–15, 15–30, and 30–45 cm bulk-soil layers—resulting in 20 composite samples in total (n = 5 per compartment). Each composite replicate represented pooled material from all three quadrats rather than an independent sample from a single quadrat. The same five-point scheme was used for all compartments, but pooling was performed separately by compartment. The rhizosphere samples represented the 0–15 cm root zone. Because quadrat-level subsamples were pooled before analysis, quadrat could not be included as a blocking or random factor. Rhizosphere soil was collected following Edwards et al. [23]. Briefly, intact root systems were carefully excavated and vigorously shaken to remove loosely adhering soil, leaving an approximately 1 mm thick layer of soil firmly attached to the root surface. The roots with adhering soil were transferred into sterile tubes containing sterile phosphate-buffered saline (PBS) and vortexed for 15 s to detach the remaining soil. The resulting soil suspension was collected as the operational rhizosphere fraction for subsequent DNA extraction. Pooling material from the three quadrats provided sufficient rhizosphere soil for each composite replicate. The PBS-recovered suspension was used only for molecular analysis, whereas physicochemical properties were measured using a separate portion of the pooled rhizosphere soil retained before PBS processing. Five composite rhizosphere samples were obtained (n = 5). Soil samples were stored in ice boxes at low temperature and quickly transported back to the laboratory for subsequent determination of soil physicochemical properties and soil microbial community analysis [24,25].

### 2.2. Determination of Soil Physicochemical Properties

pH was measured using the potentiometric method: soil suspension was prepared at a water-to-soil ratio of 2.5:1 and measured with a portable pH meter [26]. Soil organic matter (SOM) was determined using the potassium dichromate capacity method with external heating [27]. Total nitrogen (TN) was determined using the semi-micro Kjeldahl method [28]. Hydrolyzable nitrogen (HN) was determined using the alkaline hydrolysis diffusion method, with titration using acid standard solution after constant-temperature diffusion at 40 °C for 24 h [29]. Total phosphorus (TP) was determined using the acid digestion–molybdenum antimony anti-spectrophotometric method [30]. Available phosphorus (AP) was determined using the sodium bicarbonate extraction–molybdenum antimony anti-spectrophotometric method [31]. Total potassium (TK) was determined using the acid digestion–flame photometric method [32]. Available potassium (AK) was determined using the ammonium acetate extraction–flame photometric method after oscillation extraction and filtration [33].

### 2.3. Determination of Soil Microbial Diversity

Bacterial 16S rRNA gene fragments spanning approximately 291 bp of the V4 hypervariable region were amplified with the primer pair 515F (5′-GTGCCAGCMGCCGCGGTAA-3′) and 806R (5′-GGACTACHVGGGTWTCTAAT-3′) [34,35]. For fungi, the ITS1 region was targeted using the specific primers ITS5–1737F (5′-GGAAGTAAAAGTCGTAACAAGG-3′) and ITS2-2043R (5′-GCTGCGTTCTTCATCGATGC-3′) [36,37]. PCR amplification of both bacterial and fungal targets was performed under the following cycling conditions: initial denaturation at 94 °C for 5 min; 30 cycles of denaturation at 94 °C for 30 s, annealing at 52 °C for 30 s, and extension at 72 °C for 30 s; followed by a final extension at 72 °C for 10 min. After PCR amplification and library preparation, library concentrations were quantified using a Qubit 4.0 fluorometer (Thermo Fisher Scientific, Waltham, MA, USA), and fragment-size distributions were assessed using a Qsep400 Bioanalyzer (BiOptic Inc., New Taipei City, Taiwan). Libraries that passed quality control were subjected to paired-end Illumina sequencing.

### 2.4. Microbial Community Assembly Models

The assembly mechanisms of microbial communities mainly rely on the neutral community model (NCM) and phylogenetic null models. The neutral community model is based on Hubbell’s neutral theory and evaluates the influence of stochastic processes such as dispersal limitation and ecological drift on community formation by fitting species occurrence frequency with the average relative abundance within the community [38]. The phylogenetic null model quantifies the relative importance of deterministic and stochastic processes in community assembly by comparing the phylogenetic structure of actual communities with the zero-expectation distribution generated by random permutations. The neutral community model was fitted in R version 4.3.2 (R Foundation for Statistical Computing, Vienna, Austria) using the Hmisc package version 5.1-3 [39] and custom code to compare occurrence–abundance relationships among the rhizosphere and soil-depth compartments [40]. Phylogenetic null models were implemented with picante 1.8.2 [41]. The β-nearest taxon index (βNTI) measures phylogenetic turnover relative to a null distribution: βNTI > +2 indicates heterogeneous selection, βNTI < −2 indicates homogeneous selection, and |βNTI| < 2 requires further partitioning. For |βNTI| < 2, Raup–Crick dissimilarity based on Bray–Curtis (RCbray) values > +0.95 indicate dispersal limitation, values < −0.95 indicate homogenizing dispersal, and intermediate values indicate undominated processes [42].

### 2.5. Statistical Analysis

One-way ANOVA was performed in R to test differences in soil physicochemical properties and microbial α-diversity among the four compartments, using each pooled composite sample as the experimental unit (n = 5 per compartment). Because subsamples from the three quadrats were pooled before analysis, quadrat-level variation could not be estimated or included as a blocking or random factor; the analysis therefore tested compartment differences within the sampled stand. Because this was a one-factor analysis, interaction effects were not estimated. Pairwise comparisons were conducted using the least significant difference (LSD) method in the R package agricolae (1.3-7) [43]. Community composition was visualized by principal coordinates analysis (PCoA) of Bray–Curtis dissimilarities in R version 4.3.2 (R Foundation for Statistical Computing, Vienna, Austria) [44]. Compartment effects were tested by one-factor PERMANOVA on sample-wise relative-abundance tables with 9999 permutations [45]; the supplementary verification was implemented in Python 3.12.13 with NumPy 2.3.5. Associations between environmental and microbial distance matrices were evaluated using partial Mantel tests [46]. Differential species abundance analysis was completed using the R package edgeR (version 4.0.16), which is based on the negative binomial distribution model and can effectively identify differentially enriched species between different treatment groups [47].

## 3. Results

### 3.1. Differences in Soil Physicochemical Properties Between Different Soil Layers and Rhizosphere Compartments of Common Reed

**Figure 1:**
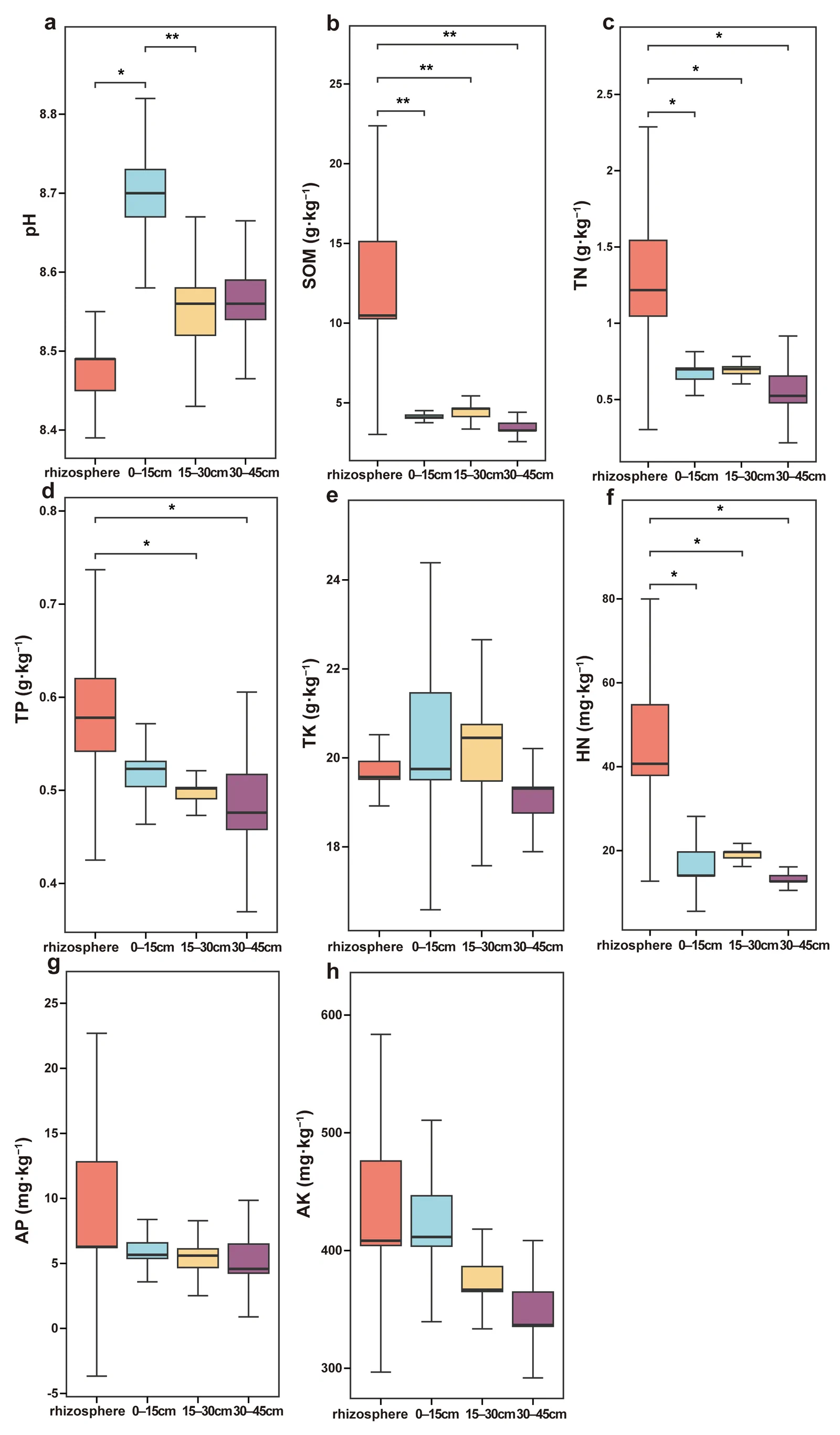
Soil physicochemical properties across bulk-soil depths and the common-reed rhizosphere. (**a**) pH; (**b**) SOM; (**c**) TN; (**d**) TP; (**e**) TK; (**f**) HN; (**g**) AP; (**h**) AK. * *p* < 0.05; ** *p* < 0.01.

Significant differences in several soil properties were detected between the rhizosphere and bulk-soil layers (*p* < 0.05 or *p* < 0.01; Figure 1). All groups remained alkaline. Mean pH was 8.712 in 0–15 cm bulk soil and 8.434 in the rhizosphere; thus, the rhizosphere showed a slight decrease in pH, not acidification (Figure 1a). SOM showed strong rhizosphere enrichment: the rhizosphere mean was 13.03 g/kg compared with 3.88–4.78 g/kg across the bulk-soil layers, and the rhizosphere differed from all three bulk-soil layers (*p* < 0.01; Figure 1b). SOM did not differ significantly among the bulk-soil layers.

TN and HN followed patterns similar to SOM (Figure 1c,f). Rhizosphere means were 1.245 g/kg for TN and 46.85 mg/kg for HN, compared with 0.551–0.717 g/kg and 14.59–19.68 mg/kg^,^ respectively, in bulk soil. These concentration differences demonstrate rhizosphere nitrogen accumulation. TP was greatest in the rhizosphere (0.577 g/kg) and differed significantly from the 15–30 and 30–45 cm layers. TK, AP, and AK did not differ significantly among compartments (Figure 1e,g,h). Any numerical depth patterns in AP or AK were therefore treated as descriptive rather than statistically supported.

### 3.2. Characteristics of Microbial α-Diversity in Different Soil Layers and Rhizosphere Compartments of Common Reed

**Figure 2:**
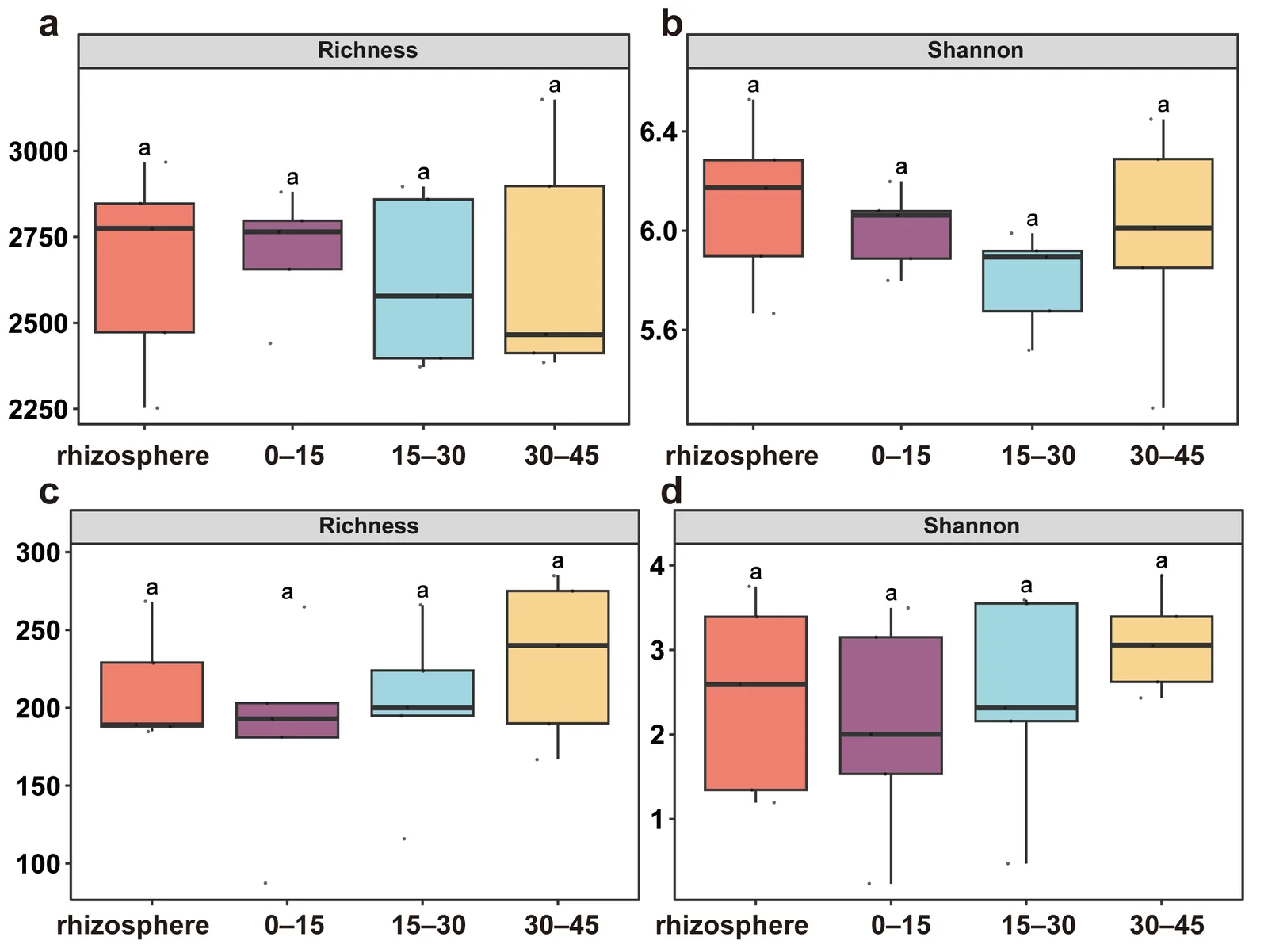
Bacterial and fungal α-diversity across bulk-soil depths and the common-reed rhizosphere. (**a**) Bacterial richness; (**b**) bacterial Shannon index; (**c**) fungal richness; (**d**) fungal Shannon index. Groups are displayed as rhizosphere, 0–15 cm, 15–30 cm, and 30–45 cm. The shared lowercase letter “a” indicates no significant differences among compartments (*p* > 0.05). The points represent five biological replicates.

Bacterial richness and Shannon diversity did not differ significantly among the rhizosphere and three bulk-soil layers (*p* > 0.05; Figure 2a,b). Mean Shannon values were 6.110 in the rhizosphere and 6.005, 5.799, and 5.977 at 0–15, 15–30, and 30–45 cm, respectively; mean richness values were 2663.0, 2707.8, 2620.4, and 2662.0 in the same order.

Fungal richness and Shannon diversity also did not differ significantly among compartments (*p* > 0.05; Figure 2c,d). Mean Shannon values were 2.454 in the rhizosphere and 2.083, 2.418, and 3.077 at 0–15, 15–30, and 30–45 cm, respectively; mean richness values were 211.8, 185.8, 200.2, and 231.4 in the same order.

### 3.3. Differences in Microbial Community Composition Between Different Soil Layers and Rhizosphere Compartments of Common Reed

**Figure 3:**
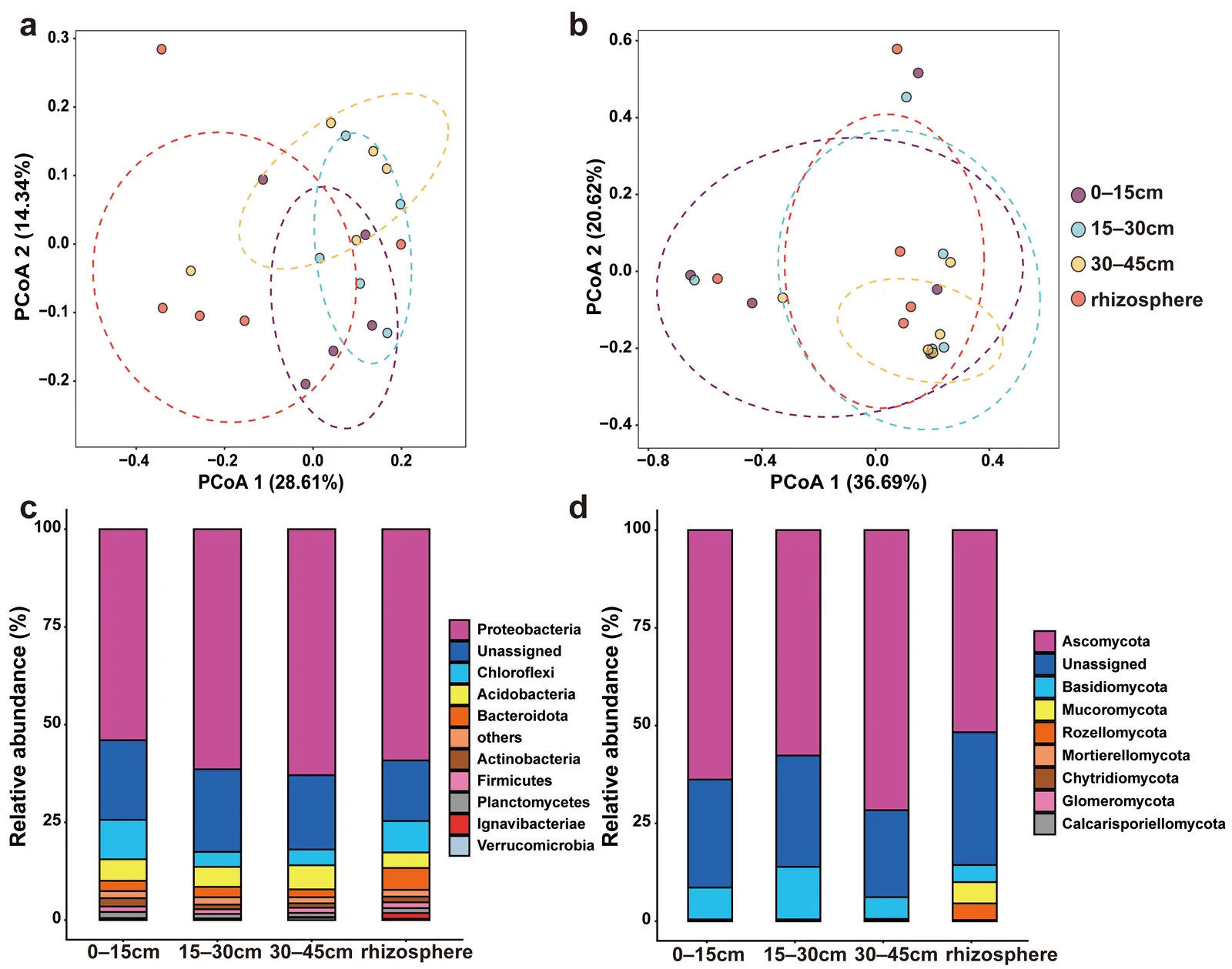
Bacterial and fungal community composition across bulk-soil depths and the common-reed rhizosphere. (**a**) PCoA of bacterial communities; (**b**) PCoA of fungal communities; (**c**) bacterial phylum-level relative abundance; (**d**) fungal phylum-level relative abundance. Groups are displayed as 0–15 cm, 15–30 cm, 30–45 cm, and rhizosphere. Relative abundance is the proportion of sequences assigned to each phylum within a compartment. PCoA represents pairwise community dissimilarities in a reduced coordinate space.

At the phylum level, bacterial communities were dominated by Proteobacteria, with relative abundance exceeding 50% in all groups (Figure 3c). Proteobacteria abundance was similar in the rhizosphere and 0–15 cm layer. Bacteroidota was relatively more abundant and Acidobacteriota relatively less abundant in the rhizosphere than in bulk soil. PCoA1 and PCoA2 explained 28.61% and 14.34% of the variation, respectively (Figure 3a), and the rhizosphere samples showed visual separation mainly along PCoA1. One-factor Bray–Curtis PERMANOVA confirmed a compartment effect on bacterial composition (pseudo-F = 1.947, R^2^ = 0.267, *p* = 0.0044).

At the phylum level, fungal communities were dominated by Ascomycota (Figure 3d). Ascomycota was relatively less abundant, whereas Mucoromycota and Rozellomycota were relatively more abundant in the rhizosphere than in the three bulk-soil layers. PCoA1 and PCoA2 explained 36.69% and 20.62% of the variation, respectively (Figure 3b), but the group clusters overlapped substantially. PERMANOVA did not detect a significant compartment effect on fungal composition (pseudo-F = 0.609, R^2^ = 0.102, *p* = 0.9112); therefore, the ordination pattern was not interpreted as confirmed microdomain differentiation.

### 3.4. Neutral-Model Characteristics of Microbial Community Assembly Across Soil Layers and the Common-Reed Rhizosphere

**Figure 4:**
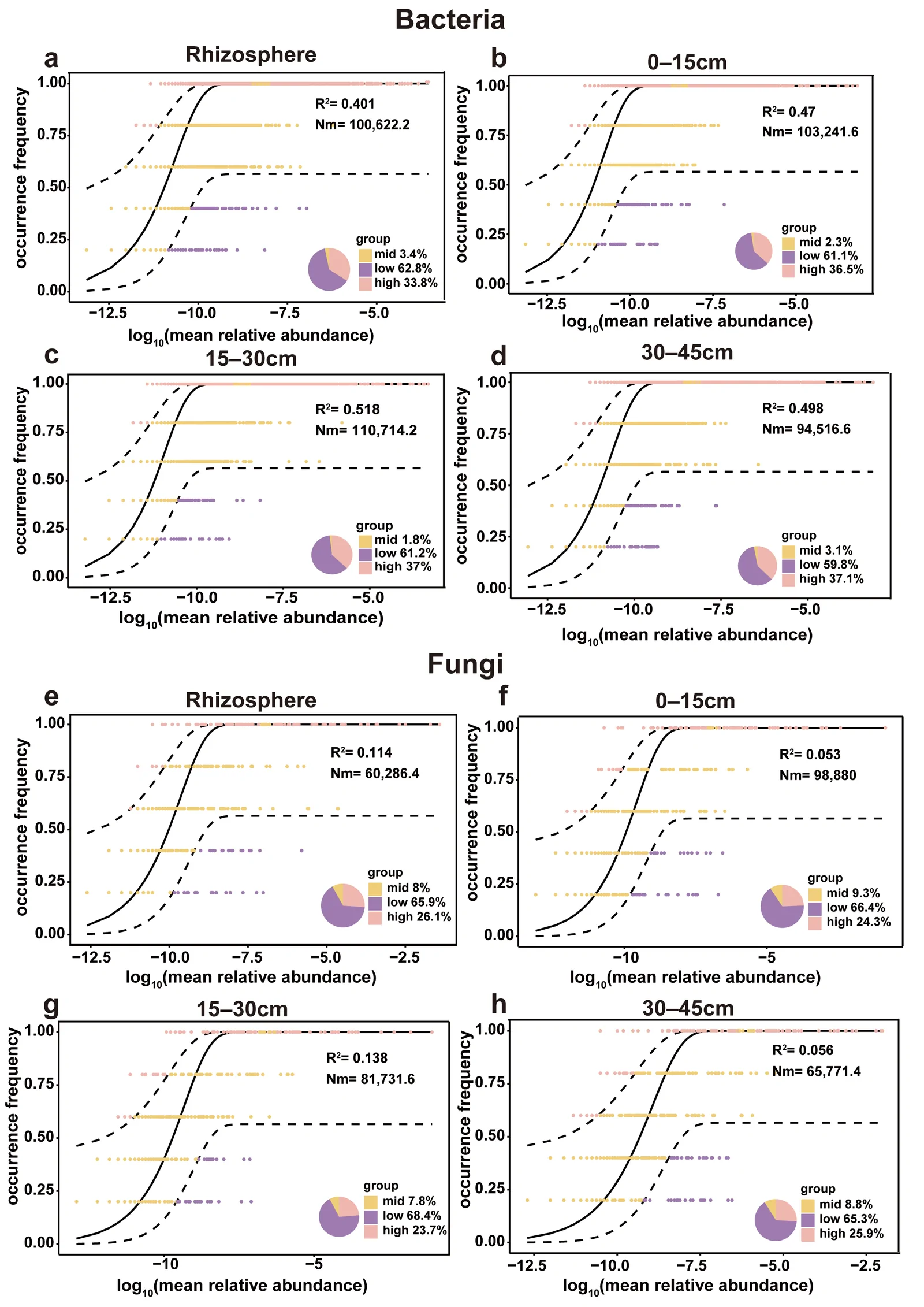
Neutral  community models of bacterial and fungal communities across bulk-soil depths and the common-reed rhizosphere. (**a**–**d**) Bacterial communities in the rhizosphere and at 0–15, 15–30, and 30–45 cm; (**e**–**h**) fungal communities in the same order. R^2^ is model fit, and Nm is the fitted migration-rate proxy.

Neutral community models were used to describe occurrence–abundance relationships and the contribution of neutral dynamics to bacterial assembly (Figure 4). Model R^2^ denotes the proportion of occurrence-frequency variation explained by the neutral model, whereas Nm is a fitted migration-rate proxy. Bacterial R^2^ and Nm values were 0.470 and 103,241.6 for 0–15 cm, 0.518 and 110,714.2 for 15–30 cm, 0.498 and 94,516.6 for 30–45 cm, and 0.401 and 100,622.2 for the rhizosphere. Thus, bacterial model fit was moderate (40.1–51.8%) and highest in the 15–30 cm layer; R^2^ alone was not treated as a direct estimate of the proportion of stochastic assembly.

Fungal neutral-model fit was poor in every compartment: R^2^ was 0.053 for 0–15 cm, 0.138 for 15–30 cm, 0.056 for 30–45 cm, and 0.114 for the rhizosphere, with corresponding Nm values of 98,880, 81,731.6, 65,771.4, and 60,286.4 (Figure 4). The model therefore explained only 5.3–13.8% of fungal occurrence-frequency variation. These low values provide limited support for inference from the fungal neutral model, so fungal assembly was interpreted primarily using the βNTI/RCbray null-model results.

### 3.5. Null-Model Validation of Microbial Community Assembly Across Soil Layers and the Common-Reed Rhizosphere

**Figure 5:**
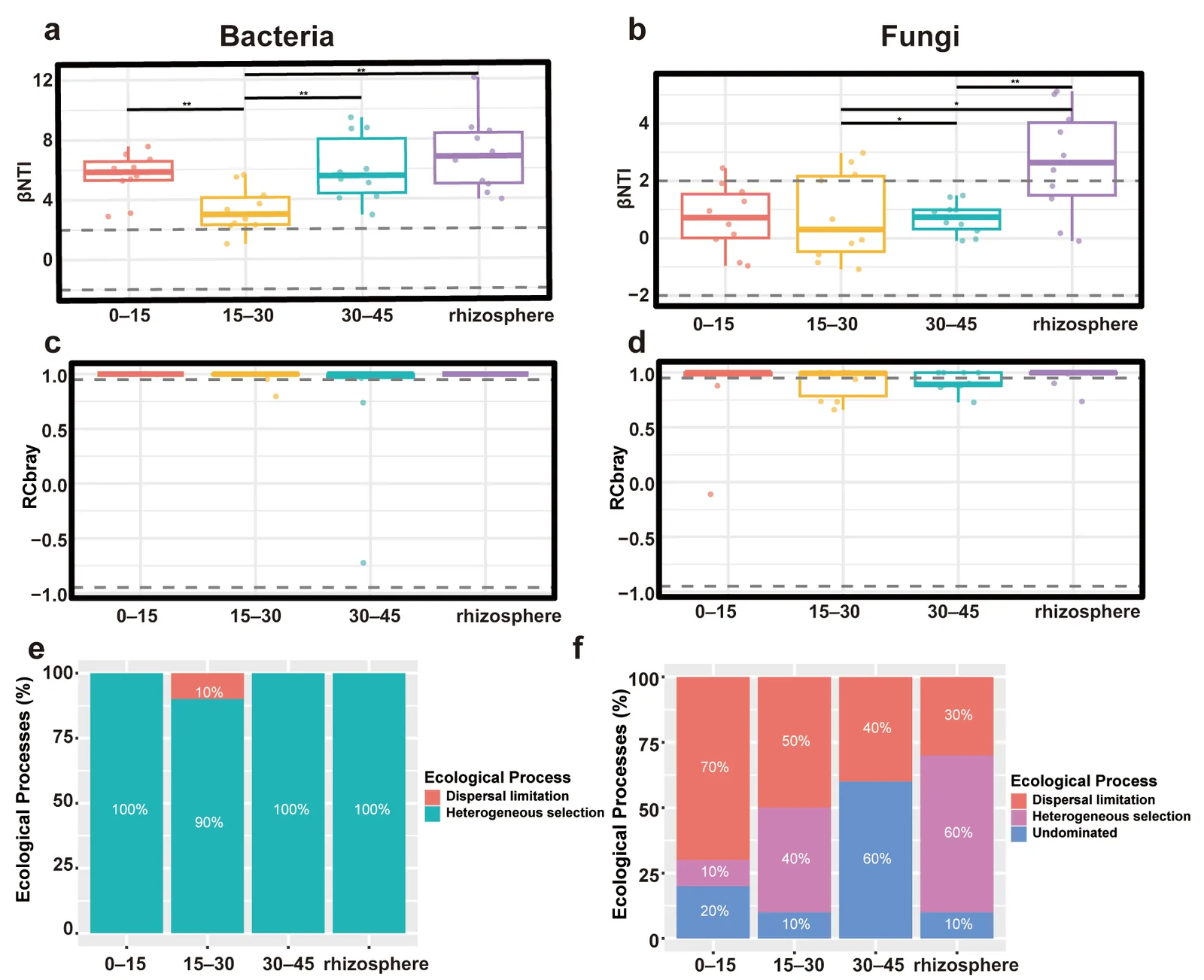
Null-model analysis of bacterial and fungal community assembly across bulk-soil depths and the common-reed rhizosphere. (**a**) Bacterial βNTI; (**b**) fungal βNTI; (**c**) bacterial RCbray; (**d**) fungal RCbray; (**e**) bacterial ecological-process proportions; (**f**) fungal ecological-process proportions. Groups are displayed as 0–15 cm, 15–30 cm, 30–45 cm, and rhizosphere. ** *p* < 0.01; * *p* < 0.05.

βNTI values for bacterial communities were generally >+2 (Figure 5a), indicating a predominance of heterogeneous selection. Significant pairwise differences occurred between 0–15 and 15–30 cm, 15–30 and 30–45 cm, and 15–30 cm and the rhizosphere. RCbray values were mainly near +1 (Figure 5c); for comparisons with |βNTI| < 2, values > +0.95 indicate dispersal limitation rather than high compositional similarity. Process partitioning showed 10% dispersal limitation and 90% heterogeneous selection in the 15–30 cm layer, whereas the other compartments were classified as 100% heterogeneous selection (Figure 5e).

Fungal assembly differed among compartments. βNTI values in the three bulk-soil layers were mainly between −2 and +2, indicating that stochastic processes required partitioning by RCbray, whereas rhizosphere βNTI values had a median > +2 and were predominantly positive (Figure 5b). Process partitioning indicated 70%, 50%, and 40% dispersal limitation in the 0–15, 15–30, and 30–45 cm layers, respectively; undominated processes accounted for 20%, 10%, and 60%, and heterogeneous selection accounted for 10%, 40%, and 0% (Figure 5f). The rhizosphere was a clear exception to the bulk-soil pattern: heterogeneous selection accounted for 60%, dispersal limitation for 30%, and undominated processes for 10%. Thus, deterministic selection predominated only in the rhizosphere, while the form of stochastic assembly varied across bulk-soil depths.

### 3.6. Correlation Analysis Between Soil Physicochemical Properties and Microbial Community Characteristics

**Figure 6:**
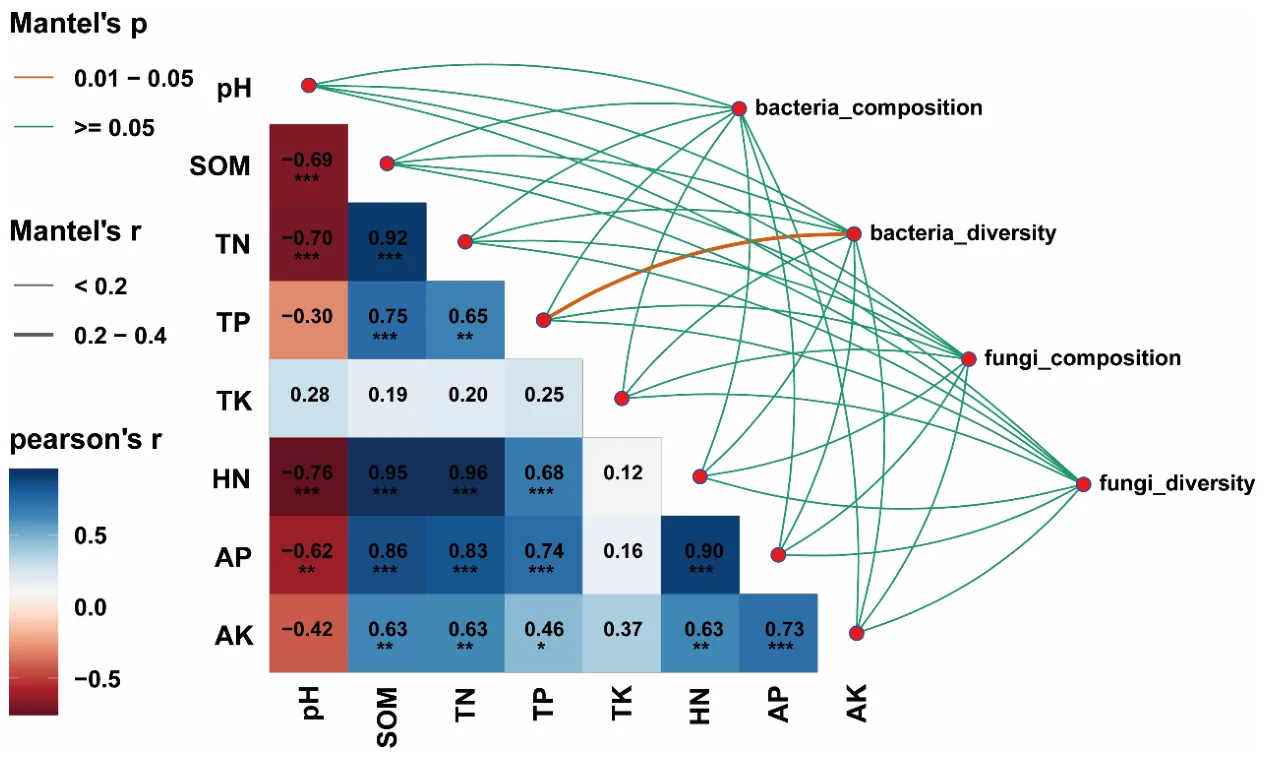
Associations between soil physicochemical properties and microbial community characteristics in common-reed soils. Partial Mantel tests evaluate correlations between environmental and microbial distance matrices. Line width is proportional to the partial Mantel statistic (thin, r < 0.2; thick, 0.2 ≤ r < 0.4), and line color indicates significance (orange, 0.01 < *p* < 0.05; green, *p* ≥ 0.05). Asterisks mark Pearson correlations among soil variables (* *p* < 0.05; ** *p* < 0.01; *** *p* < 0.001), and the color gradient represents Pearson’s r.

To evaluate associations between soil physicochemical properties and microbial attributes, Pearson correlations among soil variables and partial Mantel tests linking environmental distance matrices to community composition and α-diversity were calculated (Figure 6). SOM correlated positively with TN, HN, AP, and AK, whereas pH correlated negatively with several nutrient variables. Among the microorganism–environment Mantel links, only TP and bacterial diversity were significant (0.01 < *p* < 0.05); all links involving pH, SOM, and community composition were non-significant (*p* ≥ 0.05). Consequently, Figure 6 does not identify pH or SOM as key drivers of microbial community composition.

## 4. Discussion

### 4.1. Differentiation of Soil Physicochemical Properties and Microbial Diversity Between Rhizosphere and Non-Rhizosphere Microdomains

SOM, TN, HN, and TP in the common-reed rhizosphere were greater than in the bulk-soil layers, consistent with previous reports of rhizosphere nutrient accumulation [48]. Through root exudation, microbial recruitment, organic-matter retention, and altered nutrient transformations, the rhizosphere may develop a microenvironment distinct from bulk soil [49,50]. Contrary to the literature-based expectation of greater rhizosphere diversity, neither bacterial nor fungal richness or Shannon diversity differed significantly among compartments (*p* > 0.05), and numerical differences were not treated as evidence of a rhizosphere effect [51]. Fungal Shannon diversity was numerically highest at 30–45 cm (mean 3.077), but the compartment effect was not significant; this pattern was treated as descriptive rather than as a confirmed depth trend [52,53]. Anoxic conditions, low disturbance, and slow decomposition could potentially contribute to this numerical pattern [54]. However, redox potential, oxygen availability, and the amount and composition of recalcitrant organic matter were not measured, so these mechanisms remain hypotheses. Surface disturbance and salinity fluctuation are also possible, rather than directly demonstrated, explanations [55].

### 4.2. Vertical Shaping of Microbial Community Composition by Different Soil Depths

In bacterial communities, Proteobacteria was the absolute dominant phylum, with similar abundance in the rhizosphere and surface soil layers, potentially reflecting common-reed root activity [56,57]. The core marker of bacterial community microdomain differentiation was the increased abundance of Bacteroidota and decreased abundance of Acidobacteriota in the rhizosphere compartment. Bacteroidota is adept at utilizing easily decomposable carbon sources in root exudates and is adapted to the high-nutrient, high-oxygen environment of the rhizosphere. Acidobacteriota is more adapted to nutrient-poor, oligotrophic non-rhizosphere deep soils, with its relative abundance increasing as root influence weakens [58]. PCoA showed visual separation, and PERMANOVA confirmed a compartment effect on bacterial composition (R^2^ = 0.267, *p* = 0.0044), whereas differences among bulk-soil depths were smaller. Root exudation and oxygen release are plausible mechanisms, but they were not measured in this study [51,59].

Fungal communities were dominated by Ascomycota [60], with its abundance in the rhizosphere lower than in non-rhizosphere soil layers. Mucoromycota and Rozellomycota were relatively more abundant in the rhizosphere. The phylum-level summaries did not permit robust genus- or species-level identification or functional attribution. Accordingly, symbiotic or plant-growth-promoting roles were not inferred from phylum-level abundance alone. Fungal PCoA clusters overlapped substantially, and PERMANOVA did not detect a compartment effect (R^2^ = 0.102, *p* = 0.9112). In this dataset, compartment-level fungal compositional differences were therefore not statistically confirmed [61].

### 4.3. Microdomain Differentiation Mechanisms of Microbial Community Assembly Processes

Combining neutral- and null-model results revealed that bacterial and fungal communities in common-reed soils exhibited completely different assembly patterns, with rhizosphere effects and soil depth jointly regulating the contribution proportions of various ecological processes. Bacterial communities were overall dominated by deterministic processes, principally heterogeneous selection, with only the 15–30 cm middle soil layer showing higher contribution from stochastic processes. The elevated stochastic fraction in the 15–30 cm layer may reflect a transition in root influence and resource availability, but oxygen, root density, and resource heterogeneity were not measured. Accordingly, proposed filtering by litter, anoxia, or root exudates should be interpreted as ecological hypotheses rather than measured mechanisms [59,62]. The 15–30 cm layer had the highest bacterial Nm; any link to a relatively mild environment or resource supply remains hypothetical [63].

Fungal community assembly showed a differentiation pattern of stochastic processes in the non-rhizosphere and deterministic processes in the rhizosphere. Non-rhizosphere 0–15 cm and 15–30 cm soil layers were dominated by dispersal limitation, while the 30–45 cm deep layer was dominated by undetermined processes. The balance among stochastic processes varied with depth: dispersal limitation decreased from 70% to 50% to 40%, whereas undominated processes reached 60% at 30–45 cm [64]. Rhizosphere fungal assembly was mainly deterministic: heterogeneous selection accounted for 60%, and Nm was the lowest among compartments, a pattern consistent with strong root-associated filtering [65], although nutrient supply and microenvironment modification were not measured directly.

### 4.4. Driving Effects of Soil Physicochemical Factors on Microbial Communities

The Mantel test did not identify pH or SOM as significant drivers of bacterial or fungal community composition. All soil groups had alkaline pH values, with the highest pH in the 0–15 cm surface layer, showing a significant difference from the rhizosphere. The rhizosphere had a lower pH than 0–15 cm bulk soil but remained alkaline; any biological consequence of this modest difference requires direct testing. Organic matter showed highly significant positive correlations with total nitrogen, hydrolyzable nitrogen, available phosphorus, and available potassium, providing basic survival resources for microorganisms through the synergistic “carbon–nitrogen–phosphorus–potassium” supply. Bacterial diversity was significantly associated only with TP, but this correlation alone does not demonstrate phosphorus limitation [66]. No fungal Mantel association was statistically significant [67], and possible effects of redox conditions, organic-matter type, and carbon-source stability remain hypotheses because these variables were not measured [68]. Overall, the rhizosphere creates differentiated microenvironments by increasing carbon, nitrogen, and phosphorus nutrient contents [69], while soil layers alter oxygen supply and disturbance intensity. Together, the measured gradients were associated with bacterial composition and assembly patterns, but causal mechanisms require direct experimental tests.

### 4.5. Ecological Significance and Research Prospects

Within the sampled common-reed stand, rhizosphere effects were associated with nutrient accumulation, significant bacterial compositional differentiation, and deterministic fungal selection, whereas bulk-soil depth altered the balance among fungal assembly processes [61]. These results clarify spatial heterogeneity within one stand but do not demonstrate changes in microbial function, nutrient-cycling rates, or plant nutrient uptake. The restricted spatial design therefore limits inference beyond the sampled stand and site. Future work should combine multi-site and seasonal sampling with verified soil classification, redox potential, oxygen availability, root density, plant-tissue nutrients, root-exudate chemistry, and functional-gene or process measurements [49,50]. Such data would allow for direct tests of the mechanisms proposed here and a stronger evaluation of implications for restoration and nutrient cycling.

## 5. Conclusions

This study compared the common-reed rhizosphere with three bulk-soil depth intervals in one Yancheng coastal-wetland stand. Rhizosphere pH was lower than surface bulk-soil pH but remained alkaline, and SOM, TN, HN, and TP accumulated in the rhizosphere. No significant differences in bacterial or fungal richness or Shannon diversity were detected among compartments. Bacterial composition differed significantly by compartment (PERMANOVA R^2^ = 0.267, *p* = 0.0044), whereas fungal composition did not (R^2^ = 0.102, *p* = 0.9112). Bacterial assembly was predominantly deterministic. Fungal assembly was predominantly stochastic in bulk soil, but the rhizosphere was a clear exception, where heterogeneous selection accounted for 60% and median βNTI exceeded +2. The Mantel analysis supported only an association between TP and bacterial diversity; it did not identify pH or SOM as significant drivers of community composition. These findings should be interpreted at the scale of the sampled stand and show that rhizosphere effects and soil depth influence different components of the microbial community structure and assembly.

## Data Availability

The data presented in this study are available on request from the corresponding author due to privacy.
